# TRPV1 from the TRP family: Structure, function, implication in autoimmune diseases and potential therapies

**DOI:** 10.1080/19336950.2026.2616902

**Published:** 2026-01-22

**Authors:** Typhaine Bejoma, Yanna Pan, Qingjie Zhao

**Affiliations:** a International Education College, Shanghai University of Traditional Chinese Medicine, Shanghai, China; b State Key Laboratory of Discovery and Utilization of Functional, Components in Traditional Chinese Medicine, Innovation Research Institute of Traditional Chinese Medicine, Shanghai University of Traditional Chinese Medicine, Shanghai, China

**Keywords:** TRPV1, autoimmune disorder, ion channel, immune cells, nociceptor

## Abstract

The transient receptor potential vanilloid type 1 (TRPV1) channel, a member of the TRP ion channel family, plays a crucial role in both physiological and pathological processes. This review provides an overview of the structure, biological functions, and implications of TRPV1 in autoimmune diseases. The structural characteristics of TRPV1, including its transmembrane and intracellular domains, are examined to understand its activation and modulation. In addition to its well-known role as a thermosensor in nociceptive neurons, TRPV1 has been found to have functions in immune cells where it regulates lipid synthesis and inflammatory response. The investigation of TRPV1’s involvement in autoimmune conditions such as systemic lupus erythematosus, multiple sclerosis, and rheumatoid arthritis highlights its potential as a therapeutic target. The search for selective agonists and antagonists for TRPV1 drugs is also discussed. A comprehensive understanding of TRPV1’s structure, function, and role in autoimmune diseases lays the foundation for future studies and the development of innovative therapies targeting this channel.

## 1. Introduction

TRPV1 which stands for Transient Receptor Potential Vanilloid 1, is a member of the Transient Receptor Potential (TRP) ion channel group [1] that plays a role in the process of phototransduction [2] and is denominated following the study made by Cosens and Manning in 1969 on the *Drosophila* mutant fly that initially brought attention to the TRP’s distinctive transient functionality [3]. The TRP channels are of greatest significance in various cellular processes that are intricately linked to adaptation, homeostasis [4], and pathological conditions by their ability to detect and respond to various stimuli such as temperature, pain, pressure, and chemical signals [1,5]. The TRPV1 protein assumes a pivotal function in the regulation of pain perception, exhibiting a notable presence within the sensory neurons where it is prominently expressed [6]. In a research work led by David Julius, Michael Caterina, and colleagues, it was determined that TRPV1 is the specific nociceptor responding to the stimulation induced by a compound found in red chili pepper known as capsaicin, that causes a spicy sensation [7]. They employed a calcium-based expression cloning strategy to investigate the structure of the capsaicin receptor. Initially, a complementary DNA (cDNA) library was generated from dorsal root ganglion cells, which are sensory neurons. The cDNA library was then transfected into the HEK 293 cell line. Through this process, a clone containing a 3-kilobase cDNA insert was identified. Remarkably, this clone conferred capsaicin sensitivity, leading to the discovery of the capsaicin receptor, known as TRPV1, in 1997 [7]. TRPV1 is a nonselective cation channel abundantly expressed in a wide array of cell types distributed throughout the body [8], and it has been reported that TRPV1 plays a role in the modulation of the immune system [9–11], consequently, investigations are examining the potential role of TRPV1 in the development and progression of autoimmune disorders [12].

Autoimmune diseases comprise a diverse spectrum of disorders such as multiple sclerosis, rheumatoid arthritis and type 1 diabetes, wherein the immune system erroneously directs its assault toward the body’s own healthy cells [13]. Recent studies have indicated that autoimmune diseases exert a clear impact on approximately 10% of the population [14] drawing therefore our attention to these widely destructive disorders and consequently, growing our interest in investigating the potential influence of the TRPV1 channel on autoimmune diseases. In this review, we will concisely explore the structure and function of TRPV1. Additionally, we will discuss its potential roles in autoimmune diseases, with a specific focus on its interaction with the immune cells. Furthermore, we will explore the relevance of TRPV1 in drug development for the treatment of autoimmune disorders.

## 2. Structure of TRPV1

**Figure 1.**
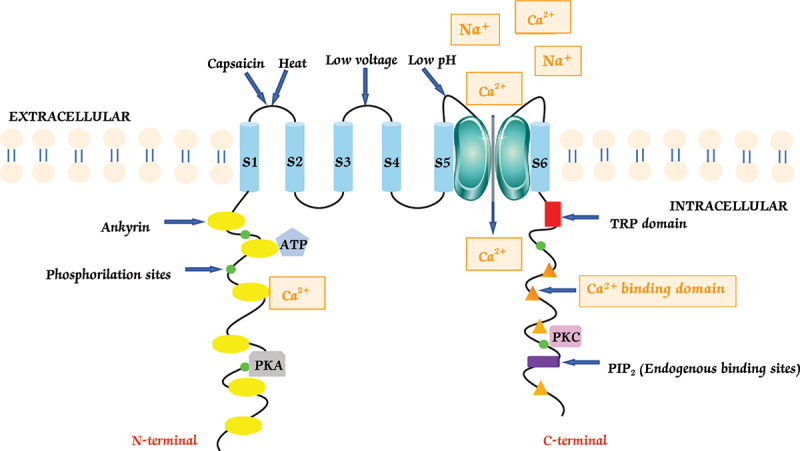
Structural architecture of the TRPV1 ion channel. Schematic diagram depicting the tetrameric structure of the TRPV1 channel. Each subunit consists of six transmembrane domains (S1-S6) that form the central ion-conducting pore. The pore loop region between S5 and S6 is critical for the selective permeability of ions such as Ca^2+^ and Na^+^. The N-terminal domain contains multiple phosphorylation sites and binding domains, while the C-terminal region includes a TRP domain and additional regulatory elements.

The TRPV1 channel has a tetrameric architecture, with four identical subunits forming a cohesive and functional channel complex. These subunits interact with each other to assemble a central pore (Figure 1) [15,16]. The TRPV1 channel has six transmembrane (TM) regions that traverse the cell membrane. These regions, designated as S1, S2, S3, S4, S5, and S6, are composed of alpha-helices [15].

Furthermore, the helices (S1-S6) span the cell membrane and connect through a pore loop domain, positioned between the fifth and sixth transmembrane segments. The S5 and S6 segments contribute to the formation of the ion-conducting pore for ions like Ca^2+^ and Na^+^. This pore loop region is crucial for selectively allowing ions to pass through the channel (Figure 1) [17].

The arrangement and interactions of the transmembrane helices, along with the pore loop domain, determine the structure and function of the TRPV1 channel (Figure 1).

TRPV1 is uniquely sensitive to changes in membrane voltage [18], which take part in regulating its responses. They are very similar to the voltage-gated ion channels, including voltage-gated sodium, calcium and potassium channels [19]. Unlike typical voltage-gated ion channels, TRPV1’s voltage sensitivity appears critical for its ability to detect and transmit pain signals. This regulation does not come from the usual voltage-sensing region, but instead from negative charges in TRPV1’s outer pore [17].

The N-terminal region of the TRPV1 channel has multiple phosphorylation sites such as the protein kinase A (PKA) sites: S502, T370, T144, S116 [20], adenosine triphosphate ATP and calmodulin binding sites located on the ankyrin repeat domain that are formed by 6 ankyrin repeats of approximately 33 amino acids, which facilitate protein-protein interactions [21].

The C-terminal region of the TRPV1 channel contains a TRP domain, binding sites for substances like phosphatidylinositol 4,5-bisphosphate (PIP2) [19], and also multiple calmodulin binding domains [22]. The amino and carboxy terminals can interact with the calmodulin molecule, and the serine (S) and threonine (T) residues can be phosphorylated by calcium calmodulin kinase II (CaMKII), PKA, and protein kinase C (PKC) [23] (Figure 1) [20].

## 3. Biological function of TRPV1

**Figure 2.**
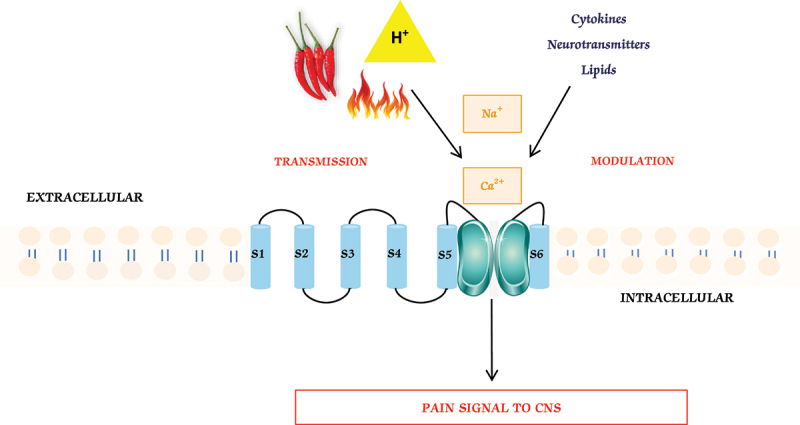
Biological function of TRPV1. Highlights of the diverse biological functions of the TRPV1 ion channel. As a thermosensor, TRPV1 responds to noxious heat stimuli, triggering the influx of calcium and sodium ions, leading to depolarization and the transmission of pain signals to the central nervous system. TRPV1 also plays a crucial role in the modulation of pain sensitivity, with phosphorylation-mediated sensitization and desensitization mechanisms. TRPV1 integrates various painful stimuli, including chemical signals, pH changes, and endogenous substances.

TRPV1 is known as a heat receptor due to its high sensitivity to heat [7]. When exposed to temperatures above a specific threshold ( >43°C), TRPV1 becomes activated, resulting in the perception of heat and pain. This heat sensitivity enables TRPV1 to function as a temperature sensor, playing a role in temperature regulation and the detection of harmful heat (Figure 2) [7].

### 3.1. Transmission

TRPV1’s pore opens between TM5 and TM6, allowing calcium and sodium ions to enter the cell [7,24,25]. This triggers depolarization and generates action potentials, transmitting pain signals to the CNS. TRPV1’s activation by noxious stimuli informs about tissue damage or harmful conditions [26]. TRPV1-expressing nociceptive neurons relay sensory information to the brain for pain perception and processing [27]. Thus, TRPV1 plays a significant role in pain transmission.

### 3.2. Modulation

TRPV1 regulates pain perception and sensitivity through sensitization and desensitization [28]. Phosphorylation of TRPV1 channels, particularly at *N*- and C-terminals (S116, T144, T370, T704, and S800), enhances sensitivity via protein PKA and PKC [20,27,29]. PKC-mediated phosphorylation increases TRPV1’s openness and pain signal transmission, while PKA phosphorylation counters desensitization, restoring responsiveness [8,20] This leads to the release of neuropeptides like substance P and CGRP [30]. TRPV1 activation is linked to hyperalgesia (increased pain reactivity) and allodynia (painful response to non-painful stimuli) [30].

### 3.3. Integration

TRPV1 is crucial in integrating diverse painful sensations, enabling perception and reaction to harmful inputs. It responds to endogenous substances like low pH, anandamide, N-oleyl-dopamine, and N-arachidonoyl-dopamine [31]. Capsaicin, an exogenous compound, selectively activates TRPV1 [8,32]. TRPV1 integrates these stimuli, contributing to pain perception, detection of noxious heat, temperature regulation, and response to chemical compounds and inflammatory mediators during injury or inflammation. TRPV1’s integration refers to its ability to detect and respond to various types of painful or noxious inputs, contributing to overall pain perception and processing.

## 4. TRPV1 and immune cells

TRPV1 is now known to be present and functional across the entire immune family (Table 1). Including macrophages, microglia, dendritic cells, mast cells, neutrophils, eosinophils, CD4^+^ and CD8^+^ T cells, regulatory T cells, B cells and innate lymphoid cells. Channel opening gives a rapid Ca^2 +^/Na^+^ pulse that tunes survival, migration, cytokine balance and effector activity, letting TRPV1 act as a context-dependent rheostat that can either amplify or resolve inflammation.

**Table 1.** TRPV1 and the immune cells.

IMMUNE CELL	TRPV1 EXPRESSION IN IMMUNE CELL	TRPV1 EFFECT	CAPSAICIN (TRPV1 AGONIST)	REFERENCE
MACROPHAGE	TNF-α Cytokine M1 macrophage	Increased secretion of TNF-α Anti-inflammatory effect	Alleviate inflammation	[33,34]
T CELL	CD4+ helper T cell fraction	Required for TCR signaling transduction in CD4+ T cells	N/A	[38,39]
B CELL	Adaptive immune response to bacterial infection	Modulate B cell immune response	N/A	[43]
DENTRIC CELL	Dermal DC But other studies were unable to identify TRPV1 in DC	Essential for the synthesis of lL-23 by dermal DC	Induce DC maturation and migration to lymph nodes	[41–43]
NEUTROPHIL	Not clearly mentioned	Modulate neutrophil function and inflammation	Induce Calcium flux in Neutrophils	[47,49]

### 4.1. Macrophages

Macrophages play a crucial role in immune processes like phagocytosis and cytokine production [33]. TRPV1 is expressed in macrophages and affects their function. In atherosclerosis, TRPV1 protects against lipid accumulation and TNF-α in macrophages [34]. TRPV1 activation in macrophages triggers pain hypersensitivity [35] and influences the tumor microenvironment [36]. In Parkinson’s disease, TRPV1 activation shifts macrophages/microglia from M1 to M2, increasing arginase-1 and CD206 while reducing iNOS and IL-6 [37]. In osteoarthritis, TRPV1 promotes a proinflammatory M1 macrophage phenotype [36].

### 4.2. T cells

T cells, including CD4+ helper cells and CD8+ cytotoxic T lymphocytes, are crucial for immune defense against infections. TRPV1 is found in CD4+ T cells, exhibiting high mRNA expression and protein localization at the plasma membrane [38]. TRPV1 is necessary for effective TCR signaling in CD4+ T cells, as demonstrated by studies on mice and humans [38,39]. TRPV1 inhibition blocks TCR-mediated signaling pathways and cytokine expression in CD4+ T cells [39]. Furthermore, TRPV1 activation in CD4+ T cells inhibits direct HIV-1 infection through calcitonin gene-related peptide (CGRP) [40].

### 4.3. B cells

B cells are essential for humoral immunity, producing antibodies to defend against infections [41]. The role of TRPV1 in B cells is not well understood. Studies using TRPV1 knockout mice showed no significant effect on B cell counts [42]. However, vagal TRPV1 neurons were found to regulate B cell populations during *Streptococcus pneumoniae* lung infection, as their removal reduced B cell infiltration [43]. Limited data exists on the TRPV1-B cell relationship. Piperine, a component of black pepper, affected B cells independently of TRPV1, suppressing activation markers and cytokine synthesis [43].

### 4.4. Dendritic cells

TRPV1 expression and functional significance in dendritic cells (DC) are uncertain and inconsistent [44]. In the context of inflammation, TRPV1+ nociceptive neurons interact with dermal DCs, influencing IL-23 synthesis [41]. Some studies suggest TRPV1 expression on DCs, with capsaicin activating DC maturation and migration [41]. However, other studies failed to detect TRPV1 expression or observe calcium influx changes in DCs treated with capsaicin [42,44]. Discrepancies may be due to dosage, duration, or cell type variations. Capsaicin may affect DCs through non-TRPV1-mediated mechanisms [43]. Recent research supports functional TRPV1 expression in DCs, macrophages, and lymphocytes, showing calcium elevation and CGRP production in response to capsaicin, dependent on TRPV1 [43].

### 4.5. Neutrophils

Neutrophils, comprising 70% of white blood cells, play a crucial role in the immune system [45]. Study reported that TRPV1 knockout mice showed increased inflammation and organ damage, with elevated neutrophil infiltration and proinflammatory cytokines [46]. Phytochemicals from *Ferula akitschkensis* [47] modify neutrophil responses via TRPV1 [48]. Neutrophils exhibit calcium fluxes upon exposure to capsaicin, and this response can be reduced by N-acetyl cysteine, a TRPV1 antagonist. In individuals with polycystic ovarian syndrome (PCOS), TRPV1 in neutrophils is involved in calcium influx, potentially contributing to the release of proinflammatory cytokines [49].

## 5. TRPV1 in autoimmune diseases

### 5.1. Autoimmune hepatitis

TRPV1 plays a role in immune system modulation, particularly in autoimmune hepatitis. Studies have shown that TRPV1 mediates the anti-inflammatory effects of cannabidiol (CBD) [50]. CBD administration decreased pro-inflammatory cytokine levels and increased the presence of suppressor cells in a hepatitis model, but these effects were abolished in TRPV1-knockout mice [50]. TRPV1 activation by CBD may also contribute to its actions in immune function [51].

### 5.2. Multiple sclerosis

In Multiple Sclerosis (MS), TRPV1 is involved in controlling neuroinflammation [52]. Genetic variations in the TRPV1 gene, specifically single-nucleotide polymorphisms (SNPs), may modulate its activity and impact neuroinflammation progression in MS [53]. The TRPV1 SNP rs222747 GG/GC genotype, associated with cortical excitability and pain modulation in MS patients, influences cerebrospinal fluid cytokine composition [53–55] However, in individuals with relapsing-remitting MS, the mRNA expression of TRPV1 and other TRP channels in peripheral blood mononuclear cells is decreased [56]. TRPV1 can be phosphorylated by calcium calmodulin-dependent kinase alpha (CaMKIIα), which plays a role in pain sensitivity and perception in MS, supporting TRPV1’s involvement in MS pain signaling [57].

### 5.3. Type 1 diabetes

TRPV1 expression is elevated in sensory nerves of the pancreas [58] and is expressed on nerves innervating islet β cells, modulating T cell function. Capsaicin administration to neonatal mice destroyed TRPV1 neurons and protected them from autoimmune diabetes. This suggests that these neurons may induce type 1 diabetes by modulating T cell proliferation and activity through substance P release, TRPV1 as a potential therapeutic target for Type 1 diabetes [59].

### 5.4. Rheumatoid arthritis

Study showed that TRPV1 knockout mice showed reduced arthritis progression after adjuvant treatment [60]. Increased TRPV1 expression in synovial fibroblasts of arthritis patients correlates with higher cytokine production [61]. TRPV1 agonists like capsaicin can alleviate arthritic pain by activating TRPV1 and inducing pain desensitization [62].

### 5.5. Systemic lupus erythematosus

TRPV1 is linked to chronic inflammatory pain and neuropeptide dysregulation in systemic lupus erythematosus (SLE). Capsaicin release of substance P increases sensitivity in SLE patients [63]. TRPV1 is expressed in endothelial colony-forming cells (ECFCs) and its activation promotes angiogenesis through anandamide uptake [64]. Optical stimulation of TRPV1 in ECFCs boosts proliferation and tube formation, potentially aiding vascular healing in SLE [65].

## 6. Therapeutic approaches

TRPV1 has been extensively studied for pain management [28,66,67]. Capsaicin, derived from chili peppers [68], is the most recognized TRPV1 activator. It is FDA-approved for shingles-associated nerve pain and has shown promise in relieving pruritus in psoriasis [69]. Other TRPV1 agonists, like resiniferatoxin (RTX) found in the Euphorbia plant, have demonstrated analgesic effects in rheumatoid arthritis [70].

**Figure 3.**
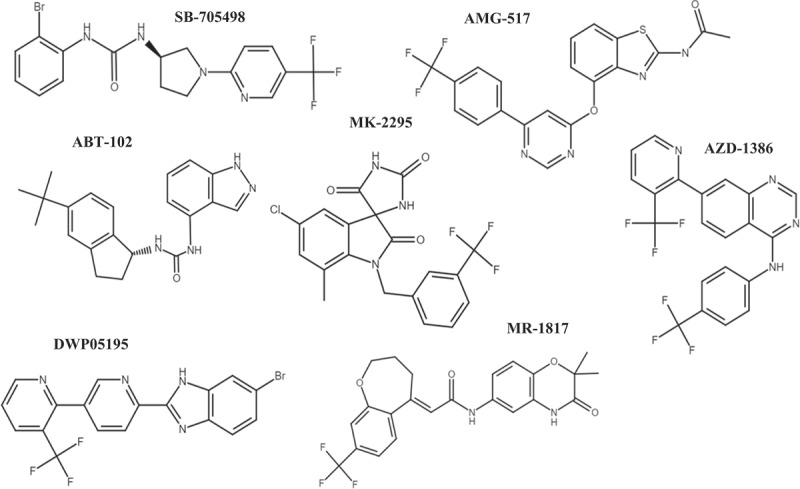
Chemical structure of TRPV1 antagonists in clinical trials. Overview of several TRPV1 antagonists that have been investigated in clinical trials for various therapeutic applications. The compounds SB-705498, AMG-517, ABT-102, MK-2295, AZD-1386, DWP05195, and MR-1817. Each of these TRPV1 antagonists has been evaluated for their potential to modulate the activity of the TRPV1 channel and their potential therapeutic benefits, particularly in the context of pain management and other clinical conditions.

Capsazepine is a competitive antagonist of capsaicin, blocking capsaicin-induced responses in dorsal root ganglion (DRG) neurons [71]. It interacts with and binds to TRPV1, specifically in the transmembrane region and the pore between transmembrane 5 and 6 (TM5, TM6) [72]. By blocking the intracellular passage of ions like Ca^2+^, which is crucial for signal transduction and immune system activation [73]. Although Capsazepine has shown therapeutic potential in conditions like cancer, hepatitis, and malaria [72], it has not undergone clinical trials and has not been specifically studied for autoimmune disorders. Several compounds, including SB-705498, AMG-517, ABT-102, MK-2295, AZD-1386, DWP05195 and MR-1817 (Figure 3), have been synthesized and tested in clinical trials as TRPV1 antagonists [72,74].

The TRPV1 antagonist SB-705498, developed by GlaxoSmithKline, was the first to undergo human testing [74]. It was evaluated as an intranasal treatment for rhinitis in Phase II trials (NCT01424397, NCT01439308) [75,76]. Additionally, it was investigated for potential therapeutic effects on migraine, pain after dental surgery, and rectal hypersensitivity in 2005. However, these trials were terminated the following year without reported data [77].

AMG-517 is a potent TRPV1 antagonist with an IC_50_ of less than 10 nM. It is available in oral form and selectively inhibits the TRPV1 channel, showing lower potency against other TRP channels (with an IC_50_ value of >5–20 µM) [78,79] However, the AMG-517 trial reported hyperthermia as an adverse effect, leading to the discontinuation of the Phase I trial [80,81].

ABT-102, developed by Abbott, is a TRPV1 antagonist that inhibits all modes of

TRPV1 activation [82]. It has been found to effectively increase body temperature in both animal models and human subjects [83,84] In a Phase I clinical trial (NCT00854659), ABT-102 administration led to an average increase of 0.6°C in core body temperature, with the highest recorded temperature reaching 38.7°C. Repeated administration of ABT-102 showed favorable tolerability, as there were no significant temperature increases observed by day 7. However, ABT-102 was also found to have a negative impact on the perception of painful heat, and this deficiency did not improve with a 7-day administration schedule [84].

The TRPV1 antagonist MK-2295, developed by Merck and Neurogen, increases thresholds for painful heat sensitivity, clinically impairing the perception of potentially harmful heat [85]. In a simulated bathing experience, participants taking MK-2295 took longer to perceive heat discomfort and remove their hands from a 49°C water bath. Furthermore, a lower percentage of MK-2295 recipients perceived 70°C water as too hot compared to the placebo group [85]. These effects were observed at doses similar to those providing pain relief.

AZD-1386 from AstraZeneca did not cause significant hyperthermia [86]. It provided acute but short-duration analgesia effects for postoperative dental removal [87]. However, in knee osteoarthritis (OA) patients, four weeks of twice-daily administration did not reduce OA discomfort [88]. The study was terminated due to elevated liver enzymes with 90 mg doses. AZD-1386 did not provide analgesia for esophageal thermal pain but increased cutaneous thermal tolerance in non-erosive gastroesophageal reflux disease (NERD) patients compared to the placebo group [88].

DWP05195, a TRPV1 antagonist synthesized by Daewoong Pharmaceutical, underwent three clinical investigations. The earliest trial (NCT00969787) assessed the safety, tolerability, pharmacokinetics, and pharmacodynamics of orally administered DWP05195 in healthy adult males [89]. The second study (NCT01094834) evaluated multiple concentrations of DWP05195 for safety, tolerability, pharmacokinetics, and pharmacodynamics in healthy adults [90]. The most recent trial (NCT01557010) investigated the efficacy and tolerability of DWP05195 in post-herpetic neuralgia patients [91]. However, no results from these trials have been reported.

MR-1817 is indicated for pain, and its initial human investigation aimed to assess its safety and tolerability [92]. However, no study data have been reported.

## 7. Conclusion

TRPV1 plays a putative role in autoimmune diseases. Its structure and biological functions, including transmission, modulation, and integration, contribute to its implication in various autoimmune disorders. TRPV1 is closely associated with different immune cells, such as macrophages, T cells, B cells, DCs, and neutrophils, further highlighting its involvement in immune responses. Autoimmune hepatitis, multiple sclerosis, type 1 diabetes, rheumatoid arthritis, and systemic lupus erythematosus are among the autoimmune diseases where TRPV1 has been implicated.

Several therapeutic approaches targeting TRPV1 have been explored, including capsaicin, capsaicin analogs like capsaizapine, and other selective TRPV1 modulators such as SB-705498, AMG-517, ABT-102, MK-2295, AZD-1386, DWP05195, and MR-1817.

Understanding the structure, function, and implications of TRPV1 in autoimmune diseases provides a foundation for future research and the development of innovative therapeutic strategies.

## Data Availability

Data sharing is not applicable to this article as no data were created or analyzed in this study.

## Abbreviations

TRPV1: the transient receptor potential vanilloid type 1
TRP: transient receptor potential
TM: transmembrane
PKA: rotein kinase A
PIP2: phosphatidylinositol 4,5-bisphosphate
ATP: adenosine triphosphate
S: serine
T: threonine
CaMKII: calcium calmodulin kinase II
PKC: protein kinase C
PCOS: polycystic ovarian syndrome
CBD: cannabidiol
MS: Multiple Sclerosis
SNPs: polymorphisms
CaMKIIα: calmodulin-dependent kinase alpha
SLE: lupus erythematosus
ECFCs: endothelial colony-forming cells
RTX: resiniferatoxin
DRG: dorsal root ganglion
OA: osteoarthritis
NERD: non-erosive gastroesophageal reflux disease
DC: dendritic cells

## References

[1] Zhang M, Ma Y, Ye X, et al. Trp (transient receptor potential) ion channel family: structures, biological functions and therapeutic interventions for diseases. Sig Transduct Target Ther. 2023;8(1):261. doi: 10.1038/s41392-023-01464-x PMC10319900 37402746

[2] Hardie RC, Juusola M. Phototransduction in Drosophila. Curr Opin Neurobiol. 2015;34:37–12. doi: 10.1016/j.conb.2015.01.008 25638280

[3] Cosens D, Manning A. Abnormal electroretinogram from a Drosophila mutant. Nature. 1969;224(5216):285–287. doi: 10.1038/224285a0 5344615

[4] Vandewauw I, De Clercq K, Mulier M, et al. A TRP channel trio mediates acute noxious heat sensing. Nature. 2018;555(7698):662–666. doi: 10.1038/nature26137 29539642

[5] Smani T, Shapovalov G, Skryma R, et al. Functional and physiopathological implications of TRP channels. Biochim Biophys Acta (BBA)-Mol Cell Res. 2015;1853(8):1772–1782. doi: 10.1016/j.bbamcr.2015.04.016 25937071

[6] Julius D. Trp channels and pain. Annual review of cell and developmental biology. Annu Rev Cell Dev Biol. 2013;29(1):355–384. doi: 10.1146/annurev-cellbio-101011-155833 24099085

[7] Caterina MJ, Schumacher MA, Tominaga M, et al. The capsaicin receptor: a heat-activated ion channel in the pain pathway. Nature. 1997;389(6653):816–824. doi: 10.1038/39807 9349813

[8] Shuba YM. Beyond neuronal heat sensing: diversity of TRPV1 heat-capsaicin receptor-channel functions. Front Cell Neurosci. 2021;14:612480. doi: 10.3389/fncel.2020.612480 33613196 PMC7892457

[9] Cremin M. Trpv1 controls innate immunity during Citrobacter rodentium enteric infection. PLoS Pathog. 2023;19(12):e1011576. 38109366 10.1371/journal.ppat.1011576 PMC10758261

[10] Majhi RK, Sahoo SS, Yadav M, et al. Functional expression of TRPV channels in T cells and their implications in immune regulation. FEBS J. 2015;282(14):2661–2681. doi: 10.1111/febs.13306 25903376

[11] Meng J, Li Y, Fischer MJM, et al. Th2 modulation of transient receptor potential channels: an unmet therapeutic intervention for atopic dermatitis. Front Immunol. 2021;12:696784. doi: 10.3389/fimmu.2021.696784 34276687 PMC8278285

[12] Qu Y, Fu Y, Liu Y, et al. The role of TRPV1 in RA pathogenesis: worthy of attention. Front Immunol. 2023;14:1232013. doi: 10.3389/fimmu.2023.1232013 37744324 PMC10514908

[13] Cuthrell KM, Tzenios N, Umber J. Burden of autoimmune disorders: a review. Asian J Immunol. 2022;6(3):1–3.

[14] Nautiyal G, Sharma I, Pandey P, et al. Autoimmune diseases: recent insights on epidemiology, pathogenesis, and prevalence rate. Artif Intel Autoimmune Dis: Appl Diagnosis, Prognosis Ther. 2024:33–58. doi:10.1007/978-981-99-9029-0_2.

[15] Cao E, Liao M, Cheng Y, et al. Trpv1 structures in distinct conformations reveal activation mechanisms. Nature. 2013;504(7478):113–118. doi: 10.1038/nature12823 24305161 PMC4023639

[16] Liao M, Cao E, Julius D, et al. Structure of the TRPV1 ion channel determined by electron cryo-microscopy. Nature. 2013;504(7478):107–112. doi: 10.1038/nature12822 24305160 PMC4078027

[17] Yang F, Xu L, Lee BH, et al. An unorthodox mechanism underlying voltage sensitivity of TRPV1 ion channel. Adv Sci. 2020;7(20):2000575. doi: 10.1002/advs.202000575 PMC7578911 33101845

[18] Voets T, Droogmans G, Wissenbach U, et al. The principle of temperature-dependent gating in cold- and heat-sensitive TRP channels. Nature. 2004;430(7001):748–754. doi: 10.1038/nature02732 15306801

[19] Yuan P. Structural biology of ThermoTRPV channels. Cell Calcium. 2019;84:102106. doi: 10.1016/j.ceca.2019.102106 31726322 PMC6893863

[20] Bhave G, Zhu W, Wang H, et al. cAMP-dependent protein kinase regulates desensitization of the capsaicin receptor (VR1) by direct phosphorylation. Neuron. 2002;35(4):721–731. doi: 10.1016/S0896-6273(02)00802-4 12194871

[21] Lishko PV, Procko E, Jin X, et al. The ankyrin repeats of TRPV1 bind multiple ligands and modulate channel sensitivity. Neuron. 2007;54(6):905–918. doi: 10.1016/j.neuron.2007.05.027 17582331

[22] García-Sanz N. Identification of a tetramerization domain in the C terminus of the vanilloid receptor. J Neurosci. 2004;24(23):5307–5314. doi: 10.1523/JNEUROSCI.0202-04.2004 15190102 PMC6729306

[23] Winter Z, Buhala A, Ötvös F, et al. Functionally important amino acid residues in the transient receptor potential vanilloid 1 (TRPV1) ion channel-an overview of the current mutational data. Mol Pain. 2013;9:1744–8069–9–30. doi: 10.1186/1744-8069-9-30 PMC3707783 23800232

[24] Tominaga M, Caterina MJ, Malmberg AB, et al. The cloned capsaicin receptor integrates multiple pain-producing stimuli. Neuron. 1998;21(3):531–543. doi: 10.1016/S0896-6273(00)80564-4 9768840

[25] Harteneck C, Plant TD, Schultz G. From worm to man: three subfamilies of TRP channels. Trends Neurosci. 2000;23(4):159–166. doi: 10.1016/S0166-2236(99)01532-5 10717675

[26] Steenland HW, Ko SW, Wu L-J, et al. Hot receptors in the brain. Mol Pain. 2006;2:1744–8069–2–34. doi: 10.1186/1744-8069-2-34 PMC1647269 17092351

[27] Immke DC, Gavva NR. The TRPV1 receptor and nociception. Semin Cell Dev Biol. 2006;17(5):582–591. doi:10.1016/j.semcdb.2006.09.004. 17196854

[28] Wang Y. The functional regulation of TRPV1 and its role in pain sensitization. Neurochem Res. 2008;33(10):2008–2012. doi: 10.1007/s11064-008-9750-5 18528757

[29] Luo L, Wang Y, Li B, et al. Molecular basis for heat desensitization of TRPV1 ion channels. Nat Commun. 2019;10(1):2134. doi: 10.1038/s41467-019-09965-6 31086183 PMC6513986

[30] Dinh QT, Groneberg DA, Peiser C, et al. Substance P expression in TRPV1 and trkA-positive dorsal root ganglion neurons innervating the mouse lung. Respir Physiol Neurobiol. 2004;144(1):15–24. doi: 10.1016/j.resp.2004.08.001 15522699

[31] Jeske NA, Diogenes A, Ruparel NB, et al. A-kinase anchoring protein mediates TRPV1 thermal hyperalgesia through PKA phosphorylation of TRPV1. Pain. 2008;138(3):604–616. doi: 10.1016/j.pain.2008.02.022 18381233 PMC2593399

[32] Abdel-Salam OM, Mózsik G. Capsaicin, the vanilloid receptor TRPV1 agonist in neuroprotection: mechanisms involved and significance. Neurochem Res. 2023;48(11):3296–3315. doi: 10.1007/s11064-023-03983-z 37493882 PMC10514110

[33] Wynn TA, Chawla A, Pollard JW. Macrophage biology in development, homeostasis and disease. Nature. 2013;496(7446):445–455. doi: 10.1038/nature12034 23619691 PMC3725458

[34] Chen O, Donnelly CR, Ji R-R. Regulation of pain by neuro-immune interactions between macrophages and nociceptor sensory neurons. Curr Opin Neurobiol. 2020;62:17–25. doi: 10.1016/j.conb.2019.11.006 31809997 PMC7266706

[35] Li Y-R, Gupta P. Immune aspects of the bi-directional neuroimmune facilitator TRPV1. Mol Biol Rep. 2019;46:1499–1510. doi:10.1007/s11033-018-4560-6. 30554315

[36] Lv Z, Xu X, Sun Z, et al. Trpv1 alleviates osteoarthritis by inhibiting M1 macrophage polarization via Ca2+/CaMKII/Nrf2 signaling pathway. Cell Death Dis. 2021;12(6):504. doi: 10.1038/s41419-021-03792-8 34006826 PMC8131608

[37] Bok E, Chung YC, Kim K-S, et al. Modulation of M1/M2 polarization by capsaicin contributes to the survival of dopaminergic neurons in the lipopolysaccharide-lesioned substantia nigra in vivo. Exp Mol Med. 2018;50(7):1–14. doi: 10.1038/s12276-018-0111-4 PMC6030094 29968707

[38] Bertin S, Aoki-Nonaka Y, de Jong PR, et al. The ion channel TRPV1 regulates the activation and proinflammatory properties of CD4+ T cells. Nat Immunol. 2014;15(11):1055–1063. doi: 10.1038/ni.3009 25282159 PMC4843825

[39] Samivel R, Kim DW, Son HR, et al. The role of TRPV1 in the CD4+ T cell-mediated inflammatory response of allergic rhinitis. Oncotarget. 2016;7(1):148. doi: 10.18632/oncotarget.6653 26700618 PMC4807989

[40] Mariotton J, Cohen E, Zhu A, et al. TRPV1 activation in human Langerhans cells and T cells inhibits mucosal HIV-1 infection via CGRP-dependent and independent mechanisms. Proc Natl Acad Sci USA. 2023;120(22):e2302509120. doi: 10.1073/pnas.2302509120 37216549 PMC10235960

[41] Basu S, Srivastava P. Immunological role of neuronal receptor vanilloid receptor 1 expressed on dendritic cells. Proc Natl Acad Sci USA. 2005;102(14):5120–5125. doi: 10.1073/pnas.0407780102 15793000 PMC555601

[42] O’Connell PJ, Pingle SC, Ahern GP. Dendritic cells do not transduce inflammatory stimuli via the capsaicin receptor TRPV1. FEBS Lett. 2005;579(23):5135–5139. doi: 10.1016/j.febslet.2005.08.023 16140298

[43] Assas MB, Wakid MH, Zakai HA, et al. Transient receptor potential vanilloid 1 expression and function in splenic dendritic cells: a potential role in immune homeostasis. Immunology. 2016;147(3):292–304. doi: 10.1111/imm.12562 26643862 PMC4754610

[44] Ghosh AK, Basu S. Tumor macrophages as a target for capsaicin mediated immunotherapy. Cancer Lett. 2012;324(1):91–97. doi: 10.1016/j.canlet.2012.05.002 22579786

[45] Actor J. Adaptive immune response and hypersensitivity. In: Elsevier’s integrated review immunology and microbiology. 2 ed. Philadelphia: Elsevier Saunders; 2012. p. 53–59.

[46] Wang Y, Wang DH. Trpv1 ablation aggravates inflammatory responses and organ damage during endotoxic shock. Clin Vaccine Immunol. 2013;20(7):1008–1015. doi: 10.1128/CVI.00674-12 23637043 PMC3697449

[47] Han R. The inhibitory effect and mechanism of Ferula akitschkensis volatile oil on gastric cancer. Evidence‐Based Comp Alternative Med. 2022;2022(1):5092742. 10.1155/2022/5092742 PMC8983199 35392643

[48] Schepetkin IA, Kushnarenko SV, Özek G, et al. Modulation of human neutrophil responses by the essential oils from Ferula akitschkensis and their constituents. J Agric Food Chem. 2016;64(38):7156–7170. doi: 10.1021/acs.jafc.6b03205 27586050 PMC5048753

[49] Köse S, Nazıroğlu M. N-acetyl cysteine reduces oxidative toxicity, apoptosis, and calcium entry through TRPV1 channels in the neutrophils of patients with polycystic ovary syndrome. Free Radic Res. 2015;49(3):338–346. doi: 10.3109/10715762.2015.1006214 25666878

[50] Hegde VL, Nagarkatti PS, Nagarkatti M. Role of myeloid-derived suppressor cells in amelioration of experimental autoimmune hepatitis following activation of TRPV1 receptors by cannabidiol. PLOS ONE. 2011;6(4):e18281. doi: 10.1371/journal.pone.0018281 21483776 PMC3069975

[51] Nichols JM, Kaplan BL. Immune responses regulated by cannabidiol. Cannabis Cannabinoid Res. 2020;5(1):12–31. doi: 10.1089/can.2018.0073 32322673 PMC7173676

[52] Stampanoni Bassi M, Gentile A, Iezzi E, et al. Transient receptor potential vanilloid 1 modulates central inflammation in multiple sclerosis. Front Neurol. 2019;10:30. doi: 10.3389/fneur.2019.00030 30761069 PMC6361812

[53] Xu H, Tian W, Fu Y, et al. Functional effects of nonsynonymous polymorphisms in the human TRPV1 gene. Am J Physiol Renal Physiol. 2007;293(6):F1865–F1876. doi: 10.1152/ajprenal.00347.2007 17913835

[54] Buttari F, Zagaglia S, Marciano L, et al. TRPV1 polymorphisms and risk of interferon β-induced flu-like syndrome in patients with relapsing-remitting multiple sclerosis. J Neuroimmunol. 2017;305:172–174. doi: 10.1016/j.jneuroim.2017.02.007 28284340

[55] Rossi S, Motta C, Studer V, et al. Tumor necrosis factor is elevated in progressive multiple sclerosis and causes excitotoxic neurodegeneration. Mult Scler. 2014;20(3):304–312. doi: 10.1177/1352458513498128 23886826

[56] Çakır M, Saçmacı H, Sabah-Özcan S. Selected transient receptor potential channel genes’ expression in peripheral blood mononuclear cells of multiple sclerosis. Hum Exp Toxicol. 2021;40(12_suppl):S406–S413. doi: 10.1177/09603271211043476 34569347

[57] Hu X, Huang F, Wang Z. CaMKIIα mechanism for pain in multiple sclerosis. J Pain. 2014;15(4):S46. doi: 10.1016/j.jpain.2014.01.191

[58] Suri A, Szallasi A. The emerging role of TRPV1 in diabetes and obesity. Trends Pharmacol Sci. 2008;29(1):29–36. doi: 10.1016/j.tips.2007.10.016 18055025

[59] Brito R, Sheth S, Mukherjea D, et al. TRPV1: a potential drug target for treating various diseases. Cells. 2014;3(2):517–545. doi: 10.3390/cells3020517 24861977 PMC4092862

[60] Fernandes ES, Russell FA, Spina D, et al. A distinct role for transient receptor potential ankyrin 1, in addition to transient receptor potential vanilloid 1, in tumor necrosis factor α–induced inflammatory hyperalgesia and Freund’s complete adjuvant–induced monarthritis. Arthritis Rheumatism. 2011;63(3):819–829. doi: 10.1002/art.30150 21360511

[61] Engler A, Aeschlimann A, Simmen BR, et al. Expression of transient receptor potential vanilloid 1 (TRPV1) in synovial fibroblasts from patients with osteoarthritis and rheumatoid arthritis. Biochem Biophys Res Commun. 2007;359(4):884–888. doi: 10.1016/j.bbrc.2007.05.178 17560936

[62] Westlund KN, Kochukov MY, Lu Y, et al. Impact of central and peripheral TRPV1 and ROS levels on proinflammatory mediators and nociceptive behavior. Mol Pain. 2010;6:1744–8069–6–46. doi: 10.1186/1744-8069-6-46 PMC2924298 20691059

[63] Sahebari M Skin reaction to capsaicin in patients with systemic lupus erythematosus compared to healthy controls. Casp J Intern Med. 2021;12(2):140. 10.22088/cjim.12.2.140 PMC8111804 34012530

[64] Lodola F, Rosti V, Tullii G, et al. Conjugated polymers optically regulate the fate of endothelial colony-forming cells. Sci Adv. 2019;5(9):eaav4620. doi: 10.1126/sciadv.aav4620 31598549 PMC6764832

[65] Komici K, Faris P, Negri S, et al. Systemic lupus erythematosus, endothelial progenitor cells and intracellular Ca2+ signaling: a novel approach for an old disease. J Autoimmun. 2020;112:102486. doi: 10.1016/j.jaut.2020.102486 32482487

[66] Jara-Oseguera A, Simon SA, Rosenbaum T. TRPV1: on the road to pain relief. Curr Mol Pharmacol. 2008;1(3):255–269. doi: 10.2174/1874467210801030255 20021438 PMC2802457

[67] Szallasi A, Cruz F, Geppetti P. TRPV1: a therapeutic target for novel analgesic drugs? Trends Mol Med. 2006;12(11):545–554. doi: 10.1016/j.molmed.2006.09.001 16996800

[68] Munjuluri S, Wilkerson DA, Sooch G, et al. Capsaicin and TRPV1 channels in the cardiovascular system: the role of inflammation. Cells. 2021;11(1):18. doi: 10.3390/cells11010018 35011580 PMC8750852

[69] Ellis CN, Berberian B, Sulica VI, et al. A double-blind evaluation of topical capsaicin in pruritic psoriasis. J Am Acad Dermatol. 1993;29(3):438–442. doi: 10.1016/0190-9622(93)70208-B 7688774

[70] Kissin EY, Freitas CF, Kissin I. The effects of intraarticular resiniferatoxin in experimental knee-joint arthritis. Anesth Analg. 2005;101(5):1433–1439. doi: 10.1213/01.ANE.0000180998.29890.B0 16244007 PMC1409708

[71] Sai MG. Treatment of oral cancer by a synthetic analogue of capsaicin & TRPV1 antagonist: capsazepine. J Drug Vigilance Alternative Therapies. 2021;1(4):129–134.

[72] Gomtsyan A, Brederson J-D. Clinical and preclinical experience with TRPV1 antagonists as potential analgesic agents. In Szallasi A, editor. TRP channels as therapeutic targets. Academic Press; 2015. p. 129–144. doi:10.1016/B978-0-12-420024-1.00008.

[73] Omari SA, Adams MJ, Geraghty DP. TRPV1 channels in immune cells and hematological malignancies. In Geraghty DP, Rash LD, editors. Advances in Pharmacology. Academic Press; 2017. p. 173–198. doi:10.1016/bs.apha.2017.01.002. 28528668

[74] Tsui H, Dorfman R, Salter MW, Dosch HM. The role of TRPV1 in diabetes. In Gomtsyan A, Faltynek CR, editors. Vanilloid receptor TRPV1 in drug discovery: targeting pain and other pathological disorders. Academic Press ; 2010. p. 423–448.

[75] GlaxoSmithKline. Intranasal SB-705498 in allergic rhinitis (AR) patients. ClinicalTrials.gov identifier: NCT01424397. 2023 [cited 2023 Sep 25]; Available from: https://www.clinicaltrials.gov/study/NCT01424397

[76] GlaxoSmithKline. Intranasal SB-705498 in non-allergic rhinitis patients. ClinicalTrials.gov identifier: nCT01439308. ClinicalTrials.gov identifier: nCT01439308. Updated December 01, 2016. Accessed September 25, 2023. https://www.clinicaltrials.gov/study/NCT01439308

[77] Doherty EM, Fotsch C, Bannon AW, et al. Novel vanilloid receptor-1 antagonists: 2. Structure− activity relationships of 4-oxopyrimidines leading to the selection of a clinical candidate. J Med Chem. 2007;50(15):3515–3527. doi: 10.1021/jm070190p 17585750

[78] Gavva NR, Bannon AW, Hovland DN, et al. Repeated administration of vanilloid receptor TRPV1 antagonists attenuates hyperthermia elicited by TRPV1 blockade. J Pharmacol Exp Ther. 2007;323(1):128–137. doi: 10.1124/jpet.107.125674 17652633

[79] Wong GY, Gavva NR. Therapeutic potential of vanilloid receptor TRPV1 agonists and antagonists as analgesics: recent advances and setbacks. Brain Res Rev. 2009;60(1):267–277. doi: 10.1016/j.brainresrev.2008.12.006 19150372

[80] Gavva NR, Treanor JJS, Garami A, et al. Pharmacological blockade of the vanilloid receptor TRPV1 elicits marked hyperthermia in humans. PAIN®. 2008;136(1):202–210. doi: 10.1016/j.pain.2008.01.024 18337008

[81] Surowy CS, Neelands TR, Bianchi BR, et al. (R)-(5-tert-butyl-2, 3-dihydro-1 H-inden-1-yl)-3-(1 H-indazol-4-yl)-urea (ABT-102) blocks polymodal activation of transient receptor potential vanilloid 1 receptors in vitro and heat-evoked firing of spinal dorsal horn neurons in vivo. J Pharmacol Exp Ther. 2008;326(3):879–888. doi: 10.1124/jpet.108.138511 18515644

[82] Honore P, Wismer CT, Mikusa J, et al. A-425619 [1-isoquinolin-5-yl-3-(4-trifluoromethyl-benzyl)-urea], a novel transient receptor potential type V1 receptor antagonist, relieves pathophysiological pain associated with inflammation and tissue injury in rats. J Pharmacol Exp Ther. 2005;314(1):410–421. doi: 10.1124/jpet.105.083915 15837818

[83] Rowbotham MC, Nothaft W, Duan RW, et al. Oral and cutaneous thermosensory profile of selective TRPV1 inhibition by ABT-102 in a randomized healthy volunteer trial. Pain. 2011;152(5):1192–1200. doi: 10.1016/j.pain.2011.01.051 21377273

[84] Safety AA. Tolerability and pharmacokinetic study of ABT-102 in healthy subjects. ClinicalTrials.gov identifier: nCT00854659. 2010 Nov 02 [cited 2023 Sep 25]. Available from: https://www.clinicaltrials.gov/study/NCT00854659

[85] Szallasi A, Sheta M. Targeting TRPV1 for pain relief: limits, losers and laurels. Expert Opin Investig Drugs. 2012;21(9):1351–1369. doi: 10.1517/13543784.2012.704021 22780443

[86] Quiding H, Jonzon B, Svensson O, et al. TRPV1 antagonistic analgesic effect: a randomized study of AZD1386 in pain after third molar extraction. PAIN®. 2013;154(6):808–812. doi: 10.1016/j.pain.2013.02.004 23541425

[87] Svensson O A phase ii randomized controlled trial evaluating the efficacy and safety of the TRPV1-antagonist AZD1386 in osteoarthritis of the knee. 13th World Congr Pain. 2010.

[88] Krarup A, Ny L, Åstrand M, et al. Randomised clinical trial: the efficacy of a transient receptor potential vanilloid 1 antagonist AZD1386 in human oesophageal pain. Alimentary Pharmacol Ther. 2011;33(10):1113–1122. doi: 10.1111/j.1365-2036.2011.04629.x 21410733

[89] LTD. D.P.C. Dwp05195 in healthy adult male volunteers. ClinicalTrials.gov identifier: nCT00969787. 2010 [cited 2023 Sep 25]; Available from: https://www.clinicaltrials.gov/study/NCT00969787

[90] LTD. D.P.C. A multiple dose study of DWP05195 in healthy adult subjects. ClinicalTrials.gov identifier: nCT01094834. 2011 [cited 2023 Sep 25]; Available from: https://www.clinicaltrials.gov/study/NCT01094834

[91] LTD. D.P.C. Daewoong Pharmaceutical Co. Ltd. evaluate the efficacy and safety of DWP05195 in subjects with post-herpetic neuralgia. ClinicalTrials.gov identifier: nCT01557010. 2014 [cited 2023 Sep 25]. Available from: https://www.clinicaltrials.gov/study/NCT01557

[92] Mochida Pharmaceutical Company, L. Study evaluating single ascending doses of MR1817. ClinicalTrials.gov identifier: nCT00960180. 2014 [cited 2023 Sep 25]; Available from: https://www.clinicaltrials.gov/study/NCT00960180

